# Uterine didelphys with cervical duplication: a Müllerian anomaly

**DOI:** 10.11604/pamj.2025.52.130.49333

**Published:** 2025-11-27

**Authors:** Khushboo Lalitkumar Shah, Deepti Shrivastava

**Affiliations:** 1 Department of Obstetrics and Gynaecology, Jawaharlal Nehru Medical College, Datta Meghe Institute of Higher Education and Research, Sawangi (Meghe), Wardha, India

**Keywords:** Uterine didelphys, cervical duplication, Müllerian anomaly, septate uterus, arcuate uterus, bicornuate uterus

## Image in medicine

A 30-year-old female came to the in vitro fertilisation outpatient department with complaints of infertility for 3 years after attempting conception, with no significant past medical history. Her menstrual cycle is regular, with no history of pelvic pain or abnormal bleeding. But then she reported occasional discomfort during sexual intercourse, which she attributed to the anatomical abnormality of her cervix that was diagnosed incidentally during a routine gynaecological exam. The patient's sexual history revealed normal sexual activity with her partner, and there were no known issues with male factor infertility. A transvaginal ultrasound revealed two distinct uterine horns, each with a separate endometrial stripe. The cervix was also duplicated, with two separate canals, confirming the diagnosis of uterine didelphys with cervical duplication. Uterine didelphys with cervical duplication is a rare congenital anomaly resulting from incomplete fusion of the Müllerian ducts during fetal development. This condition presents with distinct clinical features, often leading to reproductive challenges, obstetric complications, and menstrual irregularities. The rarity of this anomaly requires careful diagnostic evaluation for proper management. The case highlights the distinctive anatomical features, including two separate uterine horns, two cervices, and a longitudinal vaginal septum. The presence of two cervixes complicates obstetric care, particularly during labour and delivery, and may require tailored management strategies. The condition is often diagnosed incidentally during imaging for other gynaecological concerns.

**Figure 1:**
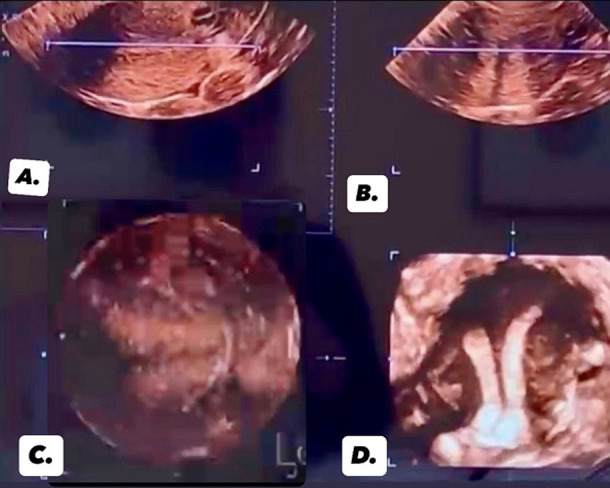
different ultrasound views for uterine didelphys: A) transverse view of uterus showing endometrial cavity in cross-section; B) sagittal view of uterus showing separation of uterine cavities; C) 3D coronal reconstruction of uterus; D) 3D coronal view of uterine cavity showing two distinct endometrial cavities, consistent with uterus didelphys

